# Supplementary material for: Targeted MRM Quantification of Urinary Proteins in Chronic Kidney Disease Caused by Glomerulopathies
Source: Molecules. 2023 Apr 9;28(8):3323. doi: 10.3390/molecules28083323 (PMC10142111; doi:10.3390/molecules28083323)
Supplement: Supplementary file 1 [file molecules-28-03323-s001.zip › Fig S1_Correlation matrix - Clinical param.pptx]

## Slide 1
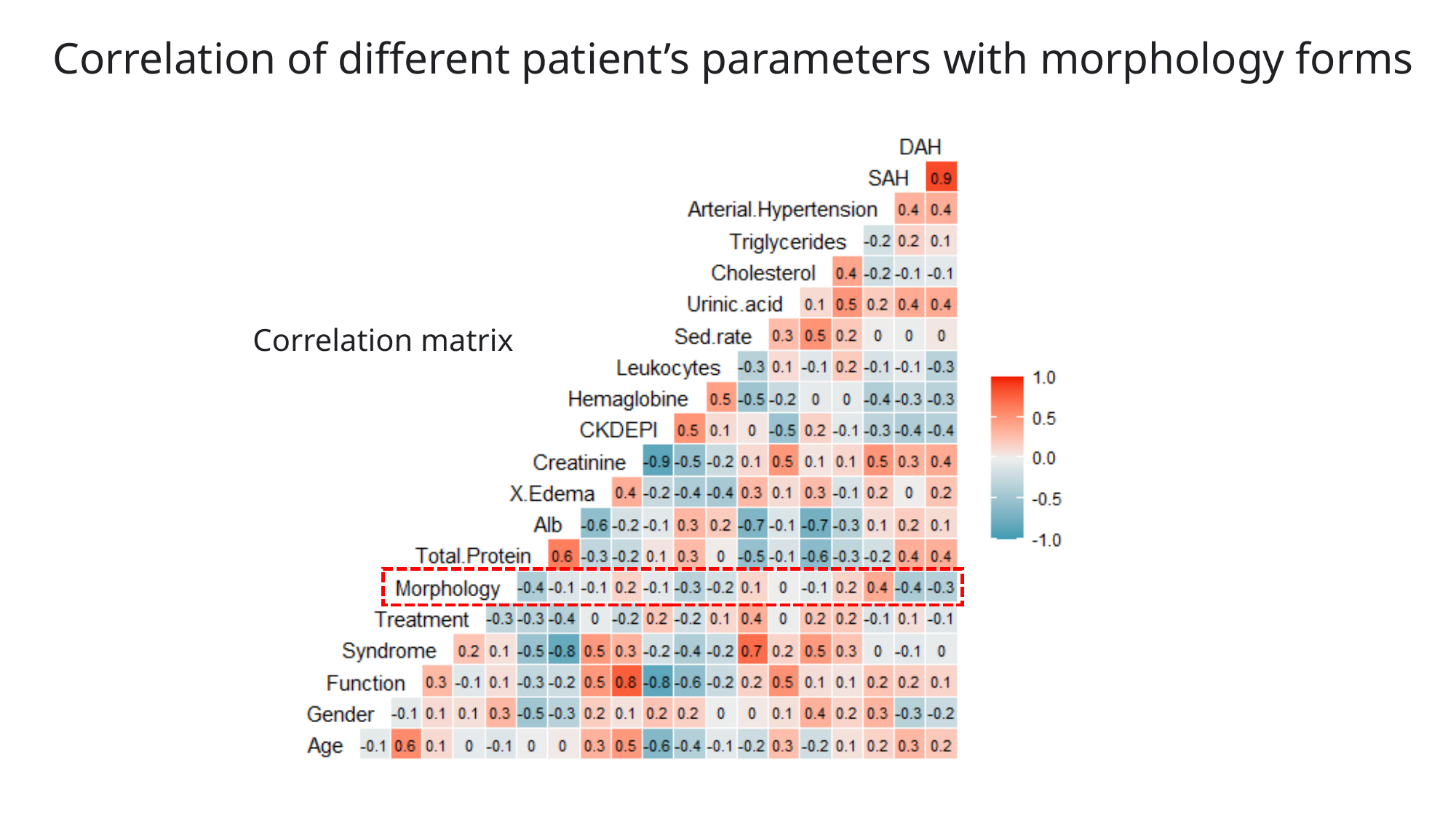

Correlation of different patient’s parameters with morphology forms
Correlation matrix
